# Clinicians’ overestimation of febrile child risk assessment

**DOI:** 10.1007/s00431-015-2667-5

**Published:** 2015-12-04

**Authors:** Evelien deVos-Kerkhof, Damian Roland, Esther de Bekker-Grob, Rianne Oostenbrink, Monica Lakhanpaul, Henriëtte A. Moll

**Affiliations:** Department of General Paediatrics, Erasmus MC-Sophia Children’s Hospital, Wytemaweg 80 room Sp-1541, 3015 CN Rotterdam, The Netherlands; Department of Health Sciences, SAPPHIRE Group, Leicester University and Leicester Hospitals, Leicester, UK; Department of Public Health, Centre for Medical Decision Making, Erasmus University Medical Centre Rotterdam, Rotterdam, The Netherlands; Department of General and Adolescent Paediatrics, UCL Institute of Child Health, Great Ormond Street, London, UK

**Keywords:** Children, Clinical prediction model, Emergency department, Fever, Serious bacterial infections

## Abstract

We aimed to estimate clinicians’ based risk thresholds at which febrile children would be managed as serious bacterial infections (SBI) to determine influencing characteristics and to compare thresholds with prediction model (Feverkidstool) risk estimates. Twenty-one video vignettes of febrile children visiting the emergency department (ED) were assessed by 42 (40.4 %) international paediatricians/paediatric emergency clinicians. Questions were related to clinical risk scores of the child having SBI and SBI management decisions on visual analogue scales. Feverkidstool risk scores were based on clinical signs/symptoms and C-reactive protein. Amongst vignettes assigned to SBI management, the median risk was 60 % (interquartile range (IQR) 30.0–80.5) and 16.0 % (IQR 5.0–32.0) when vignettes were not managed as SBI. Ill appearance and aberrant circulatory signs were the most influencing factors, as age and duration of fever were the least influencing factors on SBI management decisions. Feverkidstool risk scores varied from 13 % (IQR 7.7–28.1) for SBI management to 7.3 % (IQR 5.7–16.3) for no SBI management.

*Conclusion*: Clinicians assigned high risk scores to children who they would have managed as SBI, mostly influenced by ill appearance and aberrant circulation. In contrast to SBI risk assessment of the Feverkidstool, clinicians’ appeared to apply a more stepwise assessment of the risk of presence/absence of SBI at different steps in the diagnostic and therapeutic process. Uniform risk thresholds at which one should start SBI management in febrile children remains unclear; risk thresholds at which we refrained from SBI management were more consistent.

**Table:** 

**What is Known:**
•*Only a small proportion of febrile children presenting to the emergency department will have serious bacterial infections (SBI) and uniform risk thresholds to start or withhold SBI treatment are not known.* •*The low prevalence of SBI and consequently the low exposure of clinicians to these infections make them rely more on alarming signs or clinical decision rules.*
**What is New:**
•*Previously identified model predictors for SBI appeared to be significantly influencing factors in clinicians’ febrile child management in emergency care.* •*Clinicians’ wielded higher risk thresholds regarding SBI febrile child management than reflected by the clinical prediction model while smaller differences in risk thresholds between clinical and model prediction were observed when clinicians refrained from SBI management.*

## Introduction

The febrile child is a common presentation to emergency departments (ED) with 10 to 20 % of all paediatric patients due to febrile illness alone [14, 17, 28]. Most children suffering from simple self-limiting infections do not need treatment. However, a small proportion will have serious bacterial infections (SBI) which require investigation, hospital admission, antibiotics and in some cases intensive care admission.

Understanding health care professionals’ decision making, particularly regarding to diagnosis, treatment and follow-up is of vital importance, particularly as ED’s become increasingly overcrowded [33, 34]. Moreover, diagnostic errors, especially in infectious diseases, are amongst the most common medical misadventures of malpractice lawsuits in paediatrics [16].

To support decision making in febrile children, different clinical prediction models have been developed in the past decade [4, 7, 12, 19, 30, 31]. Although most studies on prediction models report good accuracy and high compliance, implementation in paediatric emergency care is limited. One of the reasons might be that clinicians’ intuitive estimation of probabilities may be as good as, or better than, prediction models [15, 21, 27]. Moreover, the lack of evidence on clinically based decision thresholds makes the application process of prediction models in clinical practice complex.

The aim of this study was to estimate risk thresholds at which children would be managed as SBI according to clinicians’ judgement by assessment of video vignettes of febrile children visiting the ED. Secondary measures included determining the effect of investigations by recording risk estimations after information on C-reactive protein value, determining the presenting characteristics that influence these risks and comparing clinician perceived risk with risk estimates using a validated prediction model (Feverkidstool) [19].

## Methods

### Study design and setting

We performed a cross-sectional study with real life video vignettes of febrile children who presented themselves to the children’s ED of the Leicester Royal Infirmary in Leicester, UK. All parents had given formal consent for the video images to be viewed by healthcare professionals under trust policy guidelines via previously published process [13]. Ethical consent for the collection of video images process had been granted by the National Research Ethics Committee East Midlands.

### Study population

Paediatricians and paediatric emergency clinicians from the source population of the REPEM network (Research in Paediatric Emergency Medicine, Europe; www.pemdatabase.org/REPEM.html), and Paediatricians at teaching hospitals with an interest in acute and emergency care in the Netherlands and United Kingdom, were invited (104 invitations). Non-responders were sent reminders at 4-week intervals, for a maximum of four mailings per subject.

### Study intervention—video vignettes

Twenty-one online video vignettes of febrile children were shown to the study participants. The vignettes were a mix of children in different age categories with potential SBI and children with simple self-limiting problems reflecting the different levels of severity in febrile child presentations in practice. The videos, with a mean duration of about 30 s, were originally recorded for educational purposes of paediatricians in training as part of the REMIT (Refining Evaluation Methodologies for Practice Changing Interventions) study (ISRCTN94772165). Background history and vital signs were reported as added text or could easily be interpreted from the video vignettes.

Initially, the participants were asked if they should manage the febrile child as having a SBI based on the vignette and background history (e.g. duration of fever) alone. Next, they were asked to assess the actual risk of the child having a SBI on a visual analogue scale (VAS^1^). Finally, we add different values of C-reactive protein (CRP) and asked if their risk assessment would have changed (VAS^2^). The online vignettes and the respondents were hosted on a secure password protected server.

### Data collection

All data collected online was exported in an anonymised format as an Excel file. We collected answers on the following questions: (1) Would you manage this child as having a serious bacterial infection? (Answers: yes/no). (2) Which diagnostics or therapeutics would you perform? (Options: no action and/or discharge; antipyretic; fluid trial; blood tests; chest-radiography; lumbar puncture; urine dipstick; oral antibiotics; intravenous antibiotics; admission). Study participants could tick as many items as they judged relevant. (3) What is the chance of SBI in this child? (Answer: 0–100 % on a VAS (VAS^1^)) [1]. As CRP is the strongest predictor of the Feverkidstool, we studied the additional value of CRP in clinicians’ management decision, with the following question: (4) A CRP is taken and returns at (*continuous value*) mg/l. What is the chance of SBI in this child? (Answer: 0–100 % VAS (VAS^2^)).

Participant’s background information was collected after finishing the video vignettes. These questions included (1) Are you a: Emergency Medicine clinician/Paediatrician; (2) How long have you been working as an Emergency Medicine clinician/paediatrician? (Options: <5 years; 5–10 years; 10–15 years; >15 years); (3) Have you ever missed/recognised a serious infection too late? (Options: yes/no).

### Definitions and outcome measures

All participants were informed about the predefined SBI definition in the letter for the study invitation: culture or radiographically proven bacterial infection (e.g. meningitis, sepsis, bacteremia, pneumonia, urinary tract infection, bacterial gastroenteritis, osteomyelitis or ethmoiditis). The outcome SBI in the vignettes was defined as management of the child as having a SBI.

Detailed descriptions on the Feverkidstool development and validation have been published earlier [19]. The originally reported discriminative ability according to the area under the receiver operating characteristic curve (AUC) of the model to predict pneumonia was 0.81 (standard error 0.04) and for other SBI 0.86 (standard error: 0.03) [19]. As the Feverkidstool was based on a polytomous logistic regression model, two risk scores were calculated, one for pneumonia and one for other SBI (e.g. urinary tract infection). We used the highest risk score in the comparison with the VAS risk scores of the video vignettes. We dichotomised the outcome of performed diagnostics and/or therapeutics. This outcome was scored ‘present’ if participants ticked fluid trial, blood tests, chest-radiography, lumbar puncture, urine dipstick, administration of oral/intravenous antibiotics and/or admission. When ‘no action and/or discharge and/or antipyretics’ was chosen, the outcome was scored as ‘not present’.

All vignettes had a statement on age, temperature and duration of fever. Abnormal clinical signs and symptoms were distributed amongst the different vignettes, with ten vignettes having one alarming sign, four vignettes with two alarming signs and seven vignettes having three or more alarming signs.

## Statistical analysis

First, we assessed the range of estimated median risks by clinical judgement (VAS) and the risk with the added value of CRP. Second, we measured the patient characteristics which enact SBI management with discrete choice experiment (DCE) analysis. Finally, we compared VAS risk scores with prediction model based judgement (Feverkidstool).

DCEs are a quantitative approach to assess preferences for e.g. medical interventions and are increasingly used in health care [10]. In DCEs, it is assumed that important items influencing medical interventions, such as vital signs, can be described by its characteristics (i.e. attributes) [24]. Those characteristics are further specified by variants of that characteristics (i.e. attribute levels). A second assumption is that the levels of those attributes are determined by the individuals’ preference for a medical intervention [24]. We studied the clinical variables of the Feverkidstool (www.erasmusmc.nl/feverkidstool) as attributes to the decision whether or not to manage febrile children of the vignettes as a SBI [13]. All DCE data was analysed by taking each choice amongst the two management alternatives as an observation. Using the Nlogit software http://www.limdep.com/ to the next sentence, the observations were analysed by a logit model. As there was a lack of diversity amongst the clinical variables ‘oxygen saturation’ and ‘tachypnoea’ between the vignettes, we could not analyse these variables accordingly. The variables tachycardia and prolonged capillary refill were taken together as one clinical variable as their correlation was too high. The influence of the different variable coefficients was tested for statistical significance (*p* value ≤0.05). As at this moment, no formal statistical methods to determine sample sizes for DCE exist; our study strived to reach at least 40 respondents in line with previous studies [6, 26].

## Results

Of the 104 invited participants, 50.4 % agreed to participate and 42 (40.4 %) participants finished the online video vignettes. The 42 final participants included 83 % paediatricians and 17 % paediatric emergency medicine physicians. Fifty per cent of the participants had a working experience of more than 10 years. Almost half of the participants had at least once missed or delayed recognised serious infection (Table 1).

**Table 1:** Demographics

	Participants (*n* = 42)
Specialism^a^	
Paediatric emergency medicine clinician	7 (16.7)
Paediatrician	35 (83.3)
Years of working experience^a^	
<5 years	4 (9.5)
5–10 year	17 (40.5)
10–15 years	9 (21.4)
>15 years	12 (28.6)
Missed/recognised a serious infection too late^a^	
Yes	19 (45.2)
No	23 (54.8)

^a^Absolute number (percentage)

### Study intervention—video vignettes

In Table 2, clinical characteristics of the video vignettes are summarised. Median age of the children was 12.0 months (interquartile range (IQR) 2.0–72.0), 57 % were boys and the median C-reactive protein level (CRP) was 60 mg/l (IQR 10.0–110.0). Answers on the four questions of the video vignettes are summarised in Table 3. Forty-one per cent of the video vignettes are managed as having a SBI according to the participants. Diagnostics and/or therapeutics were started in 77 % of the video vignettes. Median risk before the knowledge of CRP (VAS^1^) was 20.0 % (IQR 9.0–50.0) and with CRP information the risk (VAS^2^) increased to 30.0 % (IQR 10.0–60.0). As CRP values were already available in the first video for vignette 3 and 21, no change in risk could be measured. Details of performed diagnostics, therapeutics and follow-up are described in Table 4. More diagnostics and/or therapeutics were performed when the child was managed as SBI. Antipyretics were given in 65 % of the video vignettes with no differences when stratifying by outcome (SBI^M^). In 94 % of the video vignettes who were managed as SBI, blood tests were done and 71 % were hospitalised (Table 4).

**Table 2:** Clinical variables

	Video vignettes (*n* = 21)
Clinical variables	
Age (months)^a^	12.0 (2.0–72.0)
≤3 months	4 (19.0)
>3 months–<1 year	6 (28.6)
≥1 year–≤18 months	5 (23.8)
>18 months	6 (28.6)
Sex, male*	12 (57.1)
Temperature^a^(°C)	38.7 (38.5–40.2)
38.5–38.9 °C	12 (57.1)
39.0–39.9 °C	7 (33.3)
≥40.0 °C	2 (9.5)
Duration fever^a^ (days)	2.0 (1.0–3.0)
Prolonged capillair refill^*^ (>2 s)	4 (19.0)
Chest wall retractions^*^	3 (14.3)
Ill appearance^*^	7 (33.3)
Saturation (<94 % O_2_)^*^	1 (4.8)
Respiratory rate^a^ (/minute)	32.0 (20.0–60.0)
Tachypnoea	1 (4.8)
Heart rate^a^ (/minute)	132.0 (100.0–172.0)
Tachycardia	4 (19.0)
CRP^a^ (mg/L)	60.0 (10.0–110.0)
<40 mg/l	8 (38.1)
≥40 mg/l	7 (33.3)
≥80 mg/l	6 (28.6)
Presence of no. alarming symptoms^a^	
≤1	11 (0–1)
>1	10 (2–5)

*Absolute number (percentage)

^a^Median (min; max)

**Table 3:** Answers of 42 participants on 21 video vignettes (*n*_total_ = 882)

	Alarming symptoms	Question 1	Question 2	Question 3		Question 4
Video vignette	No.	SBI^M^	Dx/Tx*	VAS^1a^ (%)	CRP^a^ (mg/l)	VAS^2a^ (%)
1	2	3 (7.1)	16 (38.1)	10.0 (4.8–20.0)	85	26.5 (10.0–44.8)
2	1	29 (69.0)	42 (100.0)	30.0 (20.0–50.3)	70	54.5 (30.0–79.3)
3	1	11 (26.2)	26 (61.9)	16.0 (7.8–32.8)	38	10.0 (4.8–23.0)
4	3	27 (64.3)	39 (92.9)	27.0 (10.0–51.8)	100	60.0 (30.8–76.0)
5	3	41 (97.6)	42 (100.0)	81.0 (60.0–90.0)	65	71.5 (50.0–90.0)
6	3	13 (31.0)	36 (85.7)	20.5 (10.0–40.0)	90	44.0 (20.0–69.3)
7	1	23 (54.8)	33 (78.6)	–	10	30.5 (11.0–60.3)
8	1	27 (64.3)	41 (97.6)	30.0 (14.0–50.0)	25	17.0 (10.0–29.3)
9	1	4 (9.5)	25 (59.5)	10.0 (4.0 21.0)	30	9.5 (4.0–21.0)
10	2	41 (97.6)	42 (100.0)	80.0 (62.5–90.0)	50	69.5 (40.0–90.0)
11	4	9 (21.4)	38 (90.5)	10.5 (5.0–21.0)	90	40.5 (21.0–69.0)
12	1	5 (11.9)	32 (76.2)	10.5 (5.8–21.0)	28	6.0 (4.0–14.5)
13	1	0 (0)	11 (26.2)	5.0 (2.8–15.5)	36	4.0 (0.8–12.0)
14	6	16 (38.1)	38 (90.5)	16.0 (9.8–40.0)	60	30.0 (16.3–50.0)
15	3	32 (76.2)	42 (100.0)	41.5 (20.0–69.3)	75	62.5 (38.5–80.0)
16	1	1 (2.4)	15 (35.7)	8.5 (2.8–15.8)	10	1.0 (0.0–6.0)
17	3	41 (97.6)	42 (100.0)	82.5 (69.8–93. 3)	48	81.5 (49.8–91.8)
18	2	7 (16.7)	32 (76.2)	11.5 (7.8–25.3)	110	60.0 (31.0–80.0)
19	1	9 (21.4)	24 (57.1)	15.5 (8.3–30.0)	75	30.5 (19.3–50.0)
20	1	16 (38.1)	35 (83.3)	21.0 (10.0–45.5)	35	13.5 (8.0–36.3)
21	2	10 (23.8)	29 (69.0)	–	100	19.5 (6.8–30.3)
Total		365/882 (41.4)	680/882 (77.1)	20.0 (9.0–50.0)	60.0 (35.0–85.0)	30.0 (10.0–61.0)

*Absolute number (percentage); ^a^Median (25–75 percentile)

Question 1: Would you manage this child as having a serious bacterial infection?

SBI^M^: child is managed as having SBI according to participant

Question 2: Which diagnostics or therapy would you perform?

Dx/Tx: diagnostics and/ or therapy done (defined as: fluid trial; blood tests; chest-radiography; lumbar puncture; urine dipstick; administration of oral/ intravenous antibiotics or admission)

Question 3: What is the chance of SBI in this child? (Answer: 0–100 % on a VAS (VAS^1^))

VAS^1^: risk assessment *without* knowledge of CRP (0–100 % VAS)

Question 4: A CRP is taken and returns at (*continuous value*) mg/l. What is the chance of SBI in this child? (Answer: 0–100 % VAS (VAS^2^)

VAS^2^: risk assessment *with* knowledge of CRP (0–100 % VAS)

**Table 4:** Diagnostics, therapy and follow-up

Diagnostics	SBI^M^ yes *n* = 365	SBI^M^ no *n* = 517	*N*_total_ = 882
No diagnostics	4 (1.1)	100 (19.3)	104 (11.8)
Urine dipstick	252 (69.0)	134 (25.9)	386 (43.8)
Fluid trial	135 (37.0)	73 (14.1)	208 (23.6)
Blood tests	344 (94.2)	180 (34.8)	524 (59.4)
Chest-radiography	112 (30.7)	76 (14.7)	188 (21.3)
Lumbar puncture	140 (38.4)	9 (1.7)	149 (16.9)
Therapy and follow-up	SBI^M^ yes *n* = 365	SBI^M^ no *n* = 517	*N*_total_ = 882
Antipyretics	244 (66.8)	330 (63.8)	574 (65.1)
No therapy	74 (20.3)	404 (78.1)	478 (54.2)
Oral antibiotics	11 (3.0)	16 (3.1)	27 (3.1)
Intravenous antibiotics	209 (57.3)	4 (0.8)	213 (24.1)
Admission	258 (70.7)	96 (18.6)	354 (40.1)
Discharge	75 (20.5)	405 (78.3)	480 (54.4)

### Clinical judgement versus different levels of CRP

**Fig. 1:**
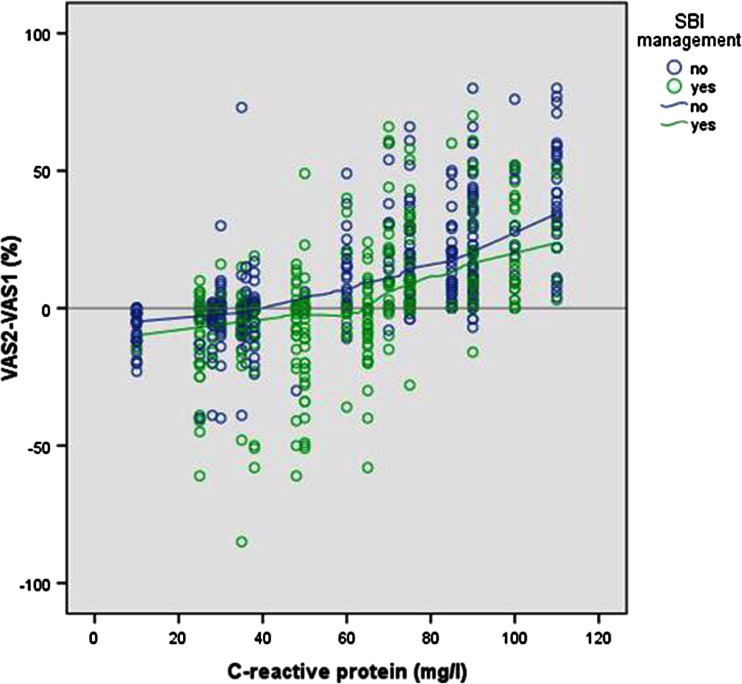
Relation video vignettes risk difference and C-reactive protein (mg/l)

In Fig. 1, the differences in clinical risk scores are visualised versus different levels of CRP values. The median clinical risk differences (VAS^2^-VAS^1^) were positively correlated with a higher level of CRP (SBI^M^ yes: Pearson correlation 0.53 (*p* = 0.000) and SBI^M^ no: Pearson correlation 0.68 (*p* = 0.000)). Risk scores of children classified initially already as being managed as SBI were influenced only by high levels of CRP (>65 mg/l), whereas children not managed initially as SBI were influenced by lower CRP levels (>40 mg/l) (Fig. 1).

### Discrete choice experiment—video vignettes

Discrete choice experiment was based upon 20 video vignettes as the clinical variables of one video were too correlated. Almost all clinical variables of the Feverkidstool could be tested with DCE analysis, except for CRP, oxygen saturation and tachypnoea. Ranking and coefficients of influencing variables on management decision of febrile children according to the DCE analysis are presented in Table 5. All tested clinical variables influenced the decision on management of febrile children significantly. Ill appearance and the combined variables of prolonged capillary refill and tachycardia were the most influencing factors and age and duration of fever the least influencing factors.

**Table 5:** Influencing variables on management decisions in febrile children (SBI^M^): a discrete choice experiment (*n*_total_ = 882)

Clinical variables	Ranking	Coefficients (SE)	*p* value
Intercept		−0.92 (0.37)	0.013
Ill appearance	1	1.15 (0.13)	<0.001
Prolonged capillary refill (>2 s) and/or tachycardia	2	0.99 (0.17)	<0.001
Chest wall retractions	3	−0.97 (0.22)	<0.001
Temperature (≥39.0 °C)	4	0.77 (0.12)	<0.001
Sex (male)	5	0.63 (0.11)	<0.001
Duration fever (days)	6	0.51 (0.20)	0.009
Age (≥1 year)	7	−0.42 (0.12)	0.001
Saturation (<94 % O_2_)	NA	NA	NA
Tachypnoea	NA	NA	NA

SBI^M^: child is managed as having SBI according to participant

*NA* not applicable, items could not been tested with DCE analyses

### Risk scores video vignettes—risk scores Feverkidstool

The median clinical risk score (VAS^2^) according to the participants amongst those video vignettes who were assigned as managed as SBI was 60.0 % (IQR 30.0–80.5) compared to a risk score according to the Feverkidstool of 12.7 % (IQR 7.7–28.1) (Table 6). When the video vignettes were not managed as SBI, the clinical risk score (VAS^2^) amounted to 16.0 % (5.0–32.0) compared to a risk of 7.3 % (5.7–16.3) according the Feverkidstool (Table 7). The largest risk score differences between the vignettes and risk scores according to the Feverkidstool were seen for video vignettes with (various levels of) decreased consciousness or agitation. This item is clearly observed when watching the video vignettes, but this clinical variable is not included in the predictors of the Feverkidstool. Finally, no differences were found in median clinical risk scores when stratified for previously missed diagnoses of the participant (*p* = 0.218).

**Table 6:** Clinical risk scores (video vignettes) versus prediction model risk scores (Feverkidstool) in children managed as SBI (SBI^M^ = yes)

	VAS^2^ (%)^a^	Feverkidstool (%)^a^
Video vignettes (no.)	SBI^M^ yes *n* = 365	*n* = 365
	Risk ≤10 %	
12	5.0 (2.0–9.5)	16.3
13	–	–
	Risk 10–50 %	
16	15.0 (15.0–15.0)	2.0
8	20.0 (12.0–30.0)	8.9
3	23.0 (9.0–61.0)	7.2
20	29.0 (12.5–61.8)	3.8
9	30.5 (8.3–66.3)	11.6
14	47.0 (32.0–76.8)	36.9
	Risk ≥50 %	
21	54.0 (17.8–80.3)	12.7
19	59.0 (45.0–90.0)	7.3
2	60.0 (30.0–80.0)	38.2
7	60.0 (30.0–72.0)	2.3
1	62.0 (50.0–62.0)	20.6
6	68.0 (35.0–83.0)	19.0
15	68.0 (52.3–80.8)	50.5
10	70.0 (44.5–90.0)	7.7
11	70.0 (57.5–81.0)	4.8
4	71.0 (35.0–80.0)	9.7
5	72.0 (50.0–90.0)	22.2
18	80.0 (21.0–82.0)	6.6
17	83.0 (50.0–92.5)	28.1
Total	60.0 (30.0–80.5)	12.7 (7.7–28.1)

**Table 7:** Clinical risk scores (video vignettes) versus prediction model risk scores (Feverkidstool) children not managed as SBI (SBI^M^ = no)

VAS^1^ (%)^a^Feverkidstool (%)^a^Video vignettes (no.)	SBI^M^ no *n* = 517	*n* = 517
	Risk ≤10 %	
16	1.0 (0.0–5.5)	2.0
13	4.0 (0.8–12.0)	5.7
12	6.0 (4.0–16.5)	16.3
9	8.5 (4.0–16.3)	11.6
3	10.0 (3.0–17.0)	7.2
8	10.0 (6.0–18.0)	8.9
20	10.0 (7.0–20.3)	3.8
	Risk 10–50 %	
10	13.0 (13.0–13.0)	7.7
21	15.5 (5.3–28.0)	12.7
7	17.0 (10.0–28.0)	2.3
1	20.0 (10.0–39.0)	20.6
14	20.0 (10.0–31.3)	36.9
17	20.0 (20.0–20.0)	28.1
19	25.0 (15.5–48.0)	7.3
11	30.0 (20.0–53.5)	4.8
5	40.0 (40.0–40.0)	22.2
6	40.0 (17.5–57.5)	19.0
2	42.0 (33.0–74.5)	38.2
4	46.0 (22.0–60.0)	9.7
15	46.5 (26.0–64.5)	50.5
	Risk ≥50 %	
18	60.0 (31.0–71.0)	6.6
Total	16.0 (5.0–32.0)	7.3 (5.7–16.3)

## Discussion

### Main findings

This is the first study on real life video vignettes to determine febrile child characteristics which enact clinicians’ management decisions. High clinical risk scores to manage febrile children as SBI were created by clinicians. All tested clinical variables of the Feverkidstool influenced clinicians’ management decisions of febrile children significantly with ill appearance and aberrant circulatory signs being the most important. Moderate CRP levels influenced risk scores in children who were initially not managed as SBI whereas high CRP levels were needed to influence risk scores in children who were initially already managed as SBI. In children managed as SBI risk thresholds judged by the clinician were higher compared with predicted risk thresholds according to the Feverkidstool. Clinical risk thresholds of children not managed as having a SBI were more comparable to prediction model-based risk thresholds.

### Comparison with literature

In this study, we aimed to get insight in patient characteristics and contextual factors influencing management decisions of the febrile child at the ED. One way to approach this process of diagnostic reasoning is decision making [11]. Decision making has been influenced by statistical models of reasoning under uncertainty using pre- and post-test probability according to Bayes’ theorem. This model deals with two major classes of errors in clinical reasoning: in the assessment of either pretest probability or the strength of the evidence [11]. Although the pretest probability of having SBI (prevalence of disease) is depending on several factors as for example age and relevant medical history, the pretest probability determined by health care setting was considered stable in the vignettes. However, we focused on the interpretation of clinicians’ strengths of evidence of the probability of a serious infection. For this decision process, we performed discrete choice experiment (DCE) analysis, which is an increasingly used method applied in studies where clinicians weigh clinical information in the diagnostic work-up [3].

In literature on diagnostic reasoning, evidence-based medicine is the most successful educational method in the translation of statistical decision theory into clinical practice [25]. Within this translation, we aimed to elaborate on the determination of quantitative decision thresholds that proved to be a complex topic. Most studies used optimised performance measures as area under the receiver operating characteristic curve (AUC) or sensitivity/specificity to establish these thresholds. Other studies described Delphi procedures to determine their clinical based cutoff points [5, 18, 20, 22, 32]. In our study, we described clinicians’ assigned median risk estimates according to which patients would have been managed as SBI. We observed agreement on clinical and prediction model-based risk thresholds when clinicians decided not to manage the febrile child as a SBI. However, the clinical risk threshold to manage the child as SBI was much higher compared with prediction model-based judgement. This phenomenon is well recognised, as clinicians don’t want to miss serious, but treatable diseases, there is a tendency to overestimate the probability of these diseases [11].

### Clinical and research implications

The most important finding of this study includes the high risk scores clinicians assigned to those children who they would have managed as SBI (median risk 60.0 % (IQR 30–80.5)). This observation is in contrast to our hypothesis that very low risk thresholds might be chosen for specific diagnosis with high morbidity/mortality (e.g. meningitis). Apparently, clinicians create more dichotomous risk estimations (high risk or low risk) for the management of specific serious infections with reassessment of risk estimates after every diagnostic step. Clinicians used a stepwise approach in the management of febrile children, rather than considering one risk thresholds for SBI in general. We observed agreement in predictive value of all tested clinical predictor variables in the detection of children with SBI, for both clinical-based as prediction model-based judgement. Clinicians were guided by ill appearance and aberrant circulatory signs in their febrile child evaluation, which were not the most influencing factors according to the Feverkidstool. For the Feverkidstool respiratory predictors as chestwall retractions and oxygen saturation were more powerful influencing factors. Furthermore, we found that CRP levels influenced clinical risk scores differently in children with or without initial SBI management, with higher influence of clinical factors than of CRP value. In our study population, this approach was not enhanced by experiences of errors in the past. These insights in influencing factors in the clinical prediction of febrile children at risk for SBI helps us to understand, review and evaluate clinical management decisions.

Compared to prediction model based risk scores, thresholds of children who were not managed as having a SBI were more comparable, ranging from 7 to 16 %. We might have to conclude that this risk threshold is justified as SBI rule-out threshold, but no agreement can be defined on rule-in thresholds as there appears too much difference between prediction model and the clinical stepwise risk assessment in children managed as SBI.

### Strengths and limitations

The main strength of this study is the use of real-life videos instead of paper-case patients. This approach is a more representative way of portraying real life, and there is an evolving evidence base on the use of patient video cases as educational interventions [8, 23].

A second strength of the study is the use of the Feverkidstool as an arithmetic model to compare the subjective overall assessment of the clinician when evaluating the febrile child. In a review describing vignette studies on medical decision behaviour, it was concluded that most studies on this topic did not compare their results to some sort of normative benchmark [3]. Moreover, the role of prediction models becomes greater, as clinicians may increasingly rely on alarming signs and symptoms described in (inter)national clinical guidelines and prediction models due to decreasing incidence of SBI. Although, there was a discrepancy in risk assessment of some video vignettes (e.g. vignettes 7, 11 and 18), probably due to the absence of variables as decreased consciousness or agitation in the Feverkidstool.

There are some other limitations in this study. Videos still lack some aspects of real life such as observation time or concise descriptions of patients’ history. However, from literature, we know that more detailed case descriptions will be assigned a higher subjective probability of disease than a brief abstract of the same case, even if they contain the same disease information [11]. Another limitation includes the determination of some clinical variables by the clinicians’ judgement (ill appearance, chestwall retractions and capillary refill time). In this way, misclassification of these clinical predictors could have occurred. However, this approach does reflect clinical practice and therefor may just strengthen generalisability of our results.

Next, the DCE analysis had to be performed within the availability of a limited number of video vignettes. As a consequence, we were forced to exclude or merge some predictor variables (e.g. oxygen saturation and tachypnoea) to meet the DCE theory design. Second, although a response rate of 50 % for clinicians was similar to other DCE studies, this response rate is not optimal [2, 9, 29]. However, due to the experienced background of all participants, we assume limited answer variability resulting in representative study results.

## Conclusion

In this study on real-life video vignettes, we observed high risk scores in clinicians’ risk estimation of SBI management in febrile children, and these risks are mostly influenced by the clinical characteristics ill appearance and aberrant circulatory signs. Uniform risk thresholds at which one should start SBI management in febrile children remains unclear, as the concept of clinicians’ dichotomous risk thresholds was hardly comparable to the overall SBI risk assessment of the prediction model. However, more consistent results were found for clinical and prediction model-based risk thresholds at which we refrain from SBI management in the febrile child visiting the emergency department.

## Abbreviations

CI: confidence interval
CRP: C-reactive protein
ED: emergency department
SBI: serious bacterial infections
VAS: visual analogue scale

## References

[1] Aitken RC (1969). Measurement of feelings using visual analogue scales. Proc R Soc Med.

[2] Ashcroft DM, Seston E, Griffiths CE (2006). Trade-offs between the benefits and risks of drug treatment for psoriasis: a discrete choice experiment with U.K. dermatologists. Br J Dermatol.

[3] Bachmann LM, Muhleisen A, Bock A, ter Riet G, Held U, Kessels AG (2008). Vignette studies of medical choice and judgement to study caregivers’ medical decision behaviour: systematic review. BMC Med Res Methodol.

[4] Bachur RG, Harper MB (2001). Predictive model for serious bacterial infections among infants younger than 3 months of age. Pediatrics.

[5] Bell LM, Grundmeier R, Localio R, Zorc J, Fiks AG, Zhang X, Stephens TB, Swietlik M, Guevara JP (2010). Electronic health record-based decision support to improve asthma care: a cluster-randomized trial. Pediatrics.

[6] Berchi C, Dupuis JM, Launoy G (2006). The reasons of general practitioners for promoting colorectal cancer mass screening in France. Eur J Health Econ.

[7] Craig JC, Williams GJ, Jones M, Codarini M, Macaskill P, Hayen A, Irwig L, Fitzgerald DA, Isaacs D, McCaskill M (2010). The accuracy of clinical symptoms and signs for the diagnosis of serious bacterial infection in young febrile children: prospective cohort study of 15 781 febrile illnesses. BMJ.

[8] D. R, T. B (2015) Using patient video cases in medical education. Arch Disd Edu Pract Accepted for publication

[9] de Bekker-Grob EW, Bliemer MC, Donkers B, Essink-Bot ML, Korfage IJ, Roobol MJ, Bangma CH, Steyerberg EW (2013). Patients’ and urologists’ preferences for prostate cancer treatment: a discrete choice experiment. Br J Cancer.

[10] de Bekker-Grob EW, Ryan M, Gerard K (2012). Discrete choice experiments in health economics: a review of the literature. Health Econ.

[11] Elstein AS, Schwartz A (2002). Clinical problem solving and diagnostic decision making: selective review of the cognitive literature. BMJ.

[12] Galetto-Lacour A, Zamora SA, Andreola B, Bressan S, Lacroix L, Da Dalt L, Gervaix A (2010). Validation of a laboratory risk index score for the identification of severe bacterial infection in children with fever without source. Arch Dis Child.

[13] Hensher D, Rose J, Green W (2005). Applied choice analysis: a primer.

[14] Kuppermann N, Fleisher GR, Jaffe DM (1998). Predictors of occult pneumococcal bacteremia in young febrile children. Ann Emerg Med.

[15] McGinn TG, Guyatt GH, Wyer PC, Naylor CD, Stiell IG, Richardson WS, Evidence-Based Medicine Working Group (2000). Users’ guides to the medical literature: XXII: how to use articles about clinical decision rules.. Jama.

[16] Najaf-Zadeh A, Dubos F, Pruvost I, Bons-Letouzey C, Amalberti R, Martinot A (2011). Epidemiology and aetiology of paediatric malpractice claims in France. Arch Dis Child.

[17] Nelson DS, Walsh K, Fleisher GR (1992). Spectrum and frequency of pediatric illness presenting to a general community hospital emergency department. Pediatrics.

[18] Nigrovic LE, Kuppermann N, Macias CG, Cannavino CR, Moro-Sutherland DM, Schremmer RD, Schwab SH, Agrawal D, Mansour KM, Bennett JE, Katsogridakis YL, Mohseni MM, Bulloch B, Steele DW, Kaplan RL, Herman MI, Bandyopadhyay S, Dayan P, Truong UT, Wang VJ, Bonsu BK, Chapman JL, Kanegaye JT, Malley R, Pediatric Emergency Medicine Collaborative Research Committee of the American Academy of Pediatrics (2007). Clinical prediction rule for identifying children with cerebrospinal fluid pleocytosis at very low risk of bacterial meningitis. JAMA.

[19] Nijman R, Vergouwe Y, Thompson M, Veen van M, Meurs Van A, Lei van der J, Steyerberg E, Moll H, Oostenbrink R (2013). Clinical prediction model to aid emergency doctors managing febrile children at risk of serious bacterial infections: diagnostic study. BMJ.

[20] Oostenbrink R, Oostenbrink JB, Moons KG, Derksen-Lubsen G, Essink-Bot ML, Grobbee DE, Redekop WK, Moll HA (2002). Cost-utility analysis of patient care in children with meningeal signs. Int J Technol Assess Health Care.

[21] Pantell RH, Newman TB, Bernzweig J, Bergman DA, Takayama JI, Segal M, Finch SA, Wasserman RC (2004). Management and outcomes of care of fever in early infancy. JAMA.

[22] Reilly BM, Evans AT, Schaider JJ, Das K, Calvin JE, Moran LA, Roberts RR, Martinez E (2002). Impact of a clinical decision rule on hospital triage of patients with suspected acute cardiac ischemia in the emergency department. Jama.

[23] Roland D, Coats T, Matheson D (2012). Towards a conceptual framework demonstrating the effectiveness of audiovisual patient descriptions (patient video cases): a review of the current literature. BMC Med Educ.

[24] Ryan M (2004). Discrete choice experiments in health care. BMJ.

[25] Sackett DL (1997). Evidence-based medicine and treatment choices. Lancet.

[26] Salkeld G, Solomon M, Butow P, Short L (2005). Discrete-choice experiment to measure patient preferences for the surgical management of colorectal cancer. Br J Surg.

[27] Sim I, Gorman P, Greenes RA, Haynes RB, Kaplan B, Lehmann H, Tang PC (2001). Clinical decision support systems for the practice of evidence-based medicine. J Am Med Inform Assoc.

[28] Slater M, Krug SE (1999). Evaluation of the infant with fever without source: an evidence based approach. Emerg Med Clin North Am.

[29] Szeinbach SL, Harpe SE, Williams PB, Elhefni H (2008). Testing for allergic disease: parameters considered and test value. BMC Fam Pract.

[30] Thompson M, Coad N, Harnden A, Mayon-White R, Perera R, Mant D (2009). How well do vital signs identify children with serious infections in paediatric emergency care?. Arch Dis Child.

[31] Van den Bruel A, Aertgeerts B, Bruyninckx R, Aerts M, Buntinx F (2007). Signs and symptoms for diagnosis of serious infections in children: a prospective study in primary care. Br J Gen Pract.

[32] Wang CJ, McGlynn EA, Brook RH, Leonard CH, Piecuch RE, Hsueh SI, Schuster MA (2006). Quality-of-care indicators for the neurodevelopmental follow-up of very low birth weight children: results of an expert panel process. Pediatrics.

[33] Wolfe I, Thompson M, Gill P, Tamburlini G, Blair M, van den Bruel A, Ehrich J, Pettoello-Mantovani M, Janson S, Karanikolos M, McKee M (2013). Health services for children in western Europe. Lancet.

[34] Yen K, Gorelick MH (2007). Strategies to improve flow in the pediatric emergency department. Pediatr Emerg Care.

